# Supplementary material for: Fabrication and characterization of ceramic-polymer composite 3D scaffolds and demonstration of osteoinductive propensity with gingival mesenchymal stem cells
Source: RSC Adv. 2023 Sep 8;13(38):26967–82. doi: 10.1039/d3ra04360f (PMC10485657; doi:10.1039/d3ra04360f)
Supplement: RA-013-D3RA04360F-s001 [file RA-013-D3RA04360F-s001.pdf]

Electronic Supplementary Material (ESI) for RSC Advances.
This journal is © The Royal Society of Chemistry 2023

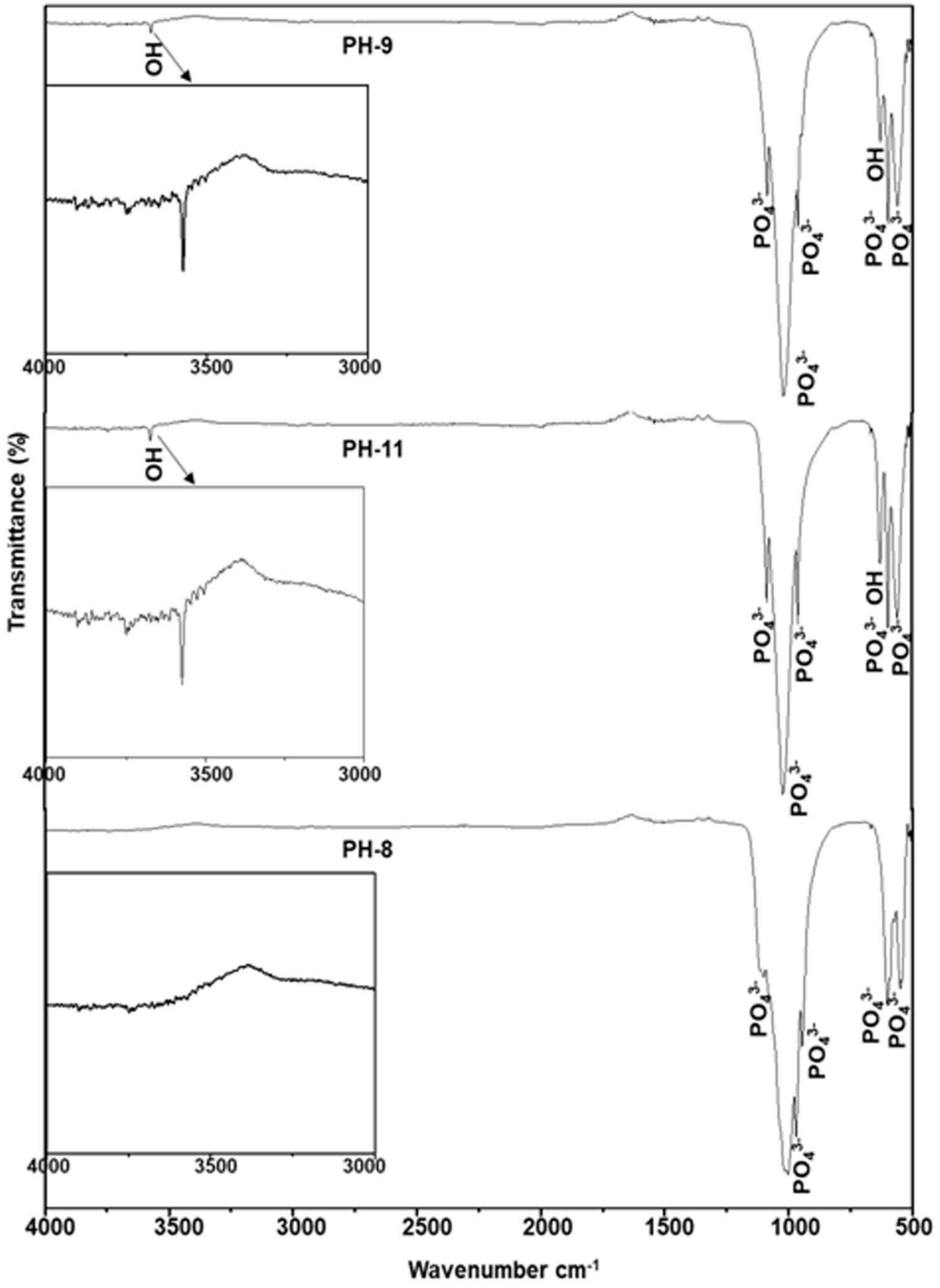

Fig. S1. Original FTIR spectra of CP nanomaterials synthesized at pH 8, 9 and 11 respectively.

a

Element	Weight%	Atomic%
O K	43.20	63.20
Na K	2.49	2.53
Mg K	0.25	0.24
P K	14.27	10.79
K K	0.20	0.12
Ca K	39.59	23.12
Totals	100.00	

Element	Weight%	Atomic%
O K	51.80	69.46
Na K	4.92	4.59
Mg K	0.80	0.70
P K	15.84	10.97
K K	0.26	0.14
Ca K	26.38	14.12
Totals	100.00	

Element	Weight%	Atomic%
O K	40.31	59.74
Na K	2.36	2.44
Mg K	0.67	0.65
P K	20.94	16.03
K K	0.27	0.16
Ca K	35.45	20.97
Totals	100.00	

b

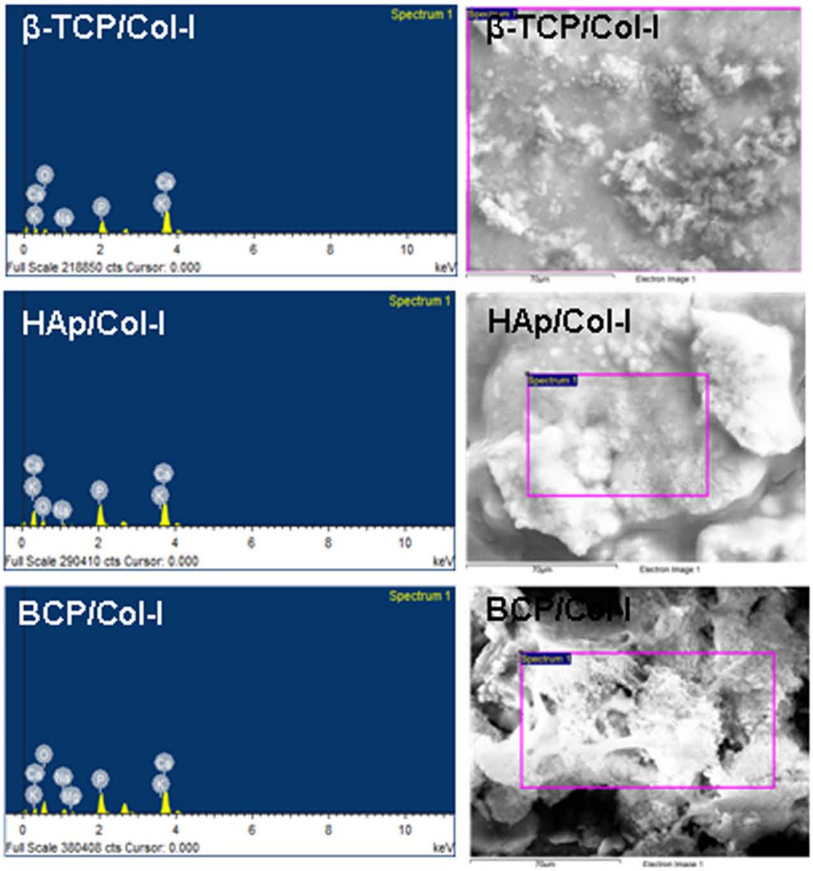

Fig. S2 (a,b). EDAX and FE-SEM of CP/Col-I scaffolds after 32 days of incubation; a) atomic % and weight % elemental present in the newly formed apatite, b) EDAX spectra and FE-SEM micrographs.
